# Axial multi-image phase retrieval under tilt illumination

**DOI:** 10.1038/s41598-017-08045-3

**Published:** 2017-08-08

**Authors:** Cheng Guo, Qiang Li, Ce Wei, Jiubin Tan, Shutian Liu, Zhengjun Liu

**Affiliations:** 1 Department of Automatic test and control, Harbin Institute of Technology, Harbin, 150080 China; 2 Department of Physics, Harbin Institute of Technology, Harbin, 150080 China

## Abstract

As a coherent diffractive imaging technique, axial multi-image phase retrieval utilizes a series of diffraction patterns on the basis of axial movement diversity to reconstruct full object wave field. Theoretically, fast convergence and high-accuracy of axial multi-image phase retrieval are demonstrated. In experiment, its retrieval suffers from the tilt illumination, in which diffraction patterns will shift in the lateral direction as the receiver traverses along the axis. In this case, the reconstructed result will be blurry or even mistaken. To solve this problem, we introduce cross-correlation calibration to derive the oblique angle and employ tilt diffraction into axial phase retrieval to recover a target, which is successfully demonstrated in simulation and experiment. Also, our method could provide a useful guidance for measuring how obliquely the incident light illuminates in an optical system.

## Introduction

Coherent diffractive imaging (CDI), as a representative lensless imaging technique, aims at reconstructing a sample in two or three dimensions by virtue of magnitude data in the diffraction filed alone without the help of reference beam^1–6^. Iterative phase retrieval algorithms with an eye to a *priori* knowledge of exit wave have been commonly applied in CDI for post-processing image synthesis^7–11^. Although generating an excellent result, these methods have been trapped in a dilemma whether or not the imposed support constraint is tight. Thus further enhancement of convergence and reconstructed accuracy has been in a slump for them.

To find a way out of stalemate, multi-image phase retrieval algorithms have been invented in the past decades^12–18^. Detecting more diffraction measurements in the CCD plane and synthesizing them algorithmically helps these methods converge to a global optimal solution. At present, multi-image phase retrieval is categorized into two modalities, namely lateral and axial scanning strategies. As a series of lateral scanning methods, the ptychographic iterative engine algorithm^13, 17, 18^ (PIE) utilizes a shifting probe to generate a number of overlapping diffraction patterns and calculates sequentially these measurement images via back and forth propagation. To simplify the experiment, single-shot ptychography^15, 16^ introduces a pinhole array to produce overlapping patterns in a single measurement. To improve the resolution of optical system, Fourier pychographic microscopy^12, 14^ (FPM) brings multi-image phase retrieval into 4-f system and reconstructs the complex amplitude by multi-angle illumination. The corresponding attempts for lateral scanning strategy have been demonstrated theoretically and experimentally. For axial scanning strategies, the single-beam multiple-intensity reconstruction technique^19^ (SBMIR) serially calculates each speckle intensity and then runs transverse propagation iteratively from one measuring plane to another, which is demonstrated by using tunable lens^20^, multiple distance^21^ and deformable mirror^22^. As a parallel method, amplitude-phase retrieval algorithm^23^ (APR) copes with each measurement image separately and it is proved to have a better reconstructed accuracy than SBMIR algorithm^24^. Free space diffraction imaging using APR algorithm has been theoretically verified in single-lens^25^ or defocused systems^26^. However, the corresponding experimental demonstration is not given, since the obliquity error of illumination hinders its ability to recover an object. Due to this reason, we propose cross-correlation calibration to inversely calculate the oblique angle and bring the tilt illumination into the axial multi-image phase retrieval model. The corresponding simulation and experiment are given to validate the performance of this scheme.

## Methods

**Figure 1:**
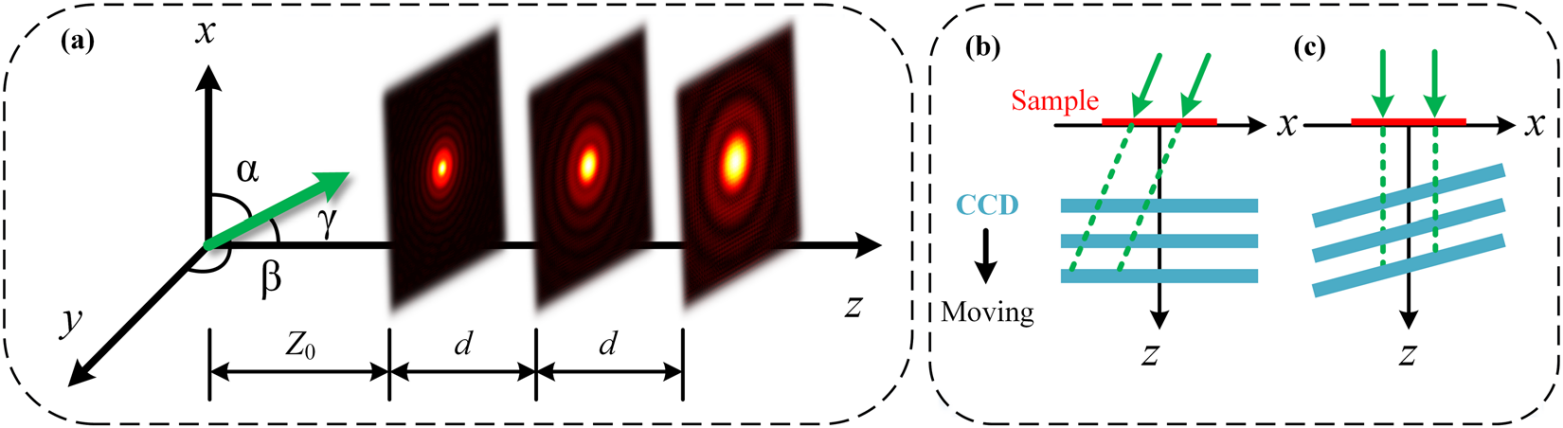
The model of tilt diffraction: (**a**) The schematic of free-space diffractive imaging model with tilt illumination. (**b**) and (**c**) denote oblique modality in the case of tilt illumination or CCD.

As an axial method, APR algorithm calculates back-and-forth iterative propagation separately and implements the average of all estimations so as to be convergent^19^. If we define APR algorithm in free-space diffraction imaging shown in Fig. 1(a), a series of diffraction patterns are taken as the amplitude constraints to retrieve a complex function located upstream. As a start, we initialize the complex amplitude of sample with zero and use the angular spectrum diffraction to simulate the propagation. Tilt illumination could lead to the shift of diffraction pattern in the lateral direction, which gives rise to a blurred retrieved image or even non-convergent result in image reconstruction.

Here we divide tilt illumination into two types based on the relative placement between CCD and sample, which are pictured in Fig. 1(b) and (c). In Fig. 1(b), the centre of diffraction pattern shifts sequentially as CCD traverses along *z* axis. In another case, the incident light illuminates vertically and CCD is placed with a tilt corresponding to *z* axis. For the case of Fig. 1(c), diffraction patterns could become distorted but not drift along *x* or *y* axis. On the contrary, the shift in Fig. 1(b) could lead to a reconstruction failure when the shift goes beyond some threshold. Hence what we focus on is the configuration in Fig. 1(b).

As is shown in Fig. 1(a), if an oblique light is defined in Cartesian coordinate with relative angles (*α*, *β*, *γ*) corresponding to *x*, *y*, *z* axes, the exit field of sample U is expressed as follows

1 $${\rm{U}}(x,y,z)={\rm{O}}(x,y,z)\,\exp [{\rm{i}}k(x\,\cos \,\alpha +y\,\cos \,\beta )]\exp ({\rm{i}}kz\,\cos \,\gamma ),$$

where the O represents object function. Here *k* = 2π/*λ* denotes wave number and *λ* is illumination wavelength. When we put sample plane in *z* = 0 and CCD plane perpendicular to *z* axis, Eq. (1) is simplified into

2 $${\rm{U}}(x,y,0)={\rm{O}}(x,y,0)\,\exp [{\rm{i}}k(x\,\cos \,\alpha +y\,\cos \,\beta )].$$

As the number of diffraction measurements is *N* and CCD plane is downstream placed at *Z*_*n*_ = *Z*_0_ + (*n* − 1)*d*, *n* ∈ [1, *N*]), from sample along *z* axis, the frequency spectrum of diffraction pattern ${{\rm{\Phi }}}_{{{\rm{Z}}}_{n}}(u,v)$ in CCD plane is presented as

3 $${{\rm{\Phi }}}_{{{\rm{Z}}}_{n}}(u,v)={\boldsymbol{ {\mathcal F} }}\{{\rm{O}}(x,y)\,\exp [{\rm{i}}k(x\,\cos \,\alpha +y\,\cos \,\beta )]\}\exp [{\rm{i}}k{{\rm{Z}}}_{n}\sqrt{1-{(\lambda u)}^{2}-{(\lambda v)}^{2}}],$$

where ${\boldsymbol{ {\mathcal F} }}$ represents Fourier transform and $\exp [{\rm{i}}k{{\rm{Z}}}_{n}\sqrt{1-{(\lambda u)}^{2}-{(\lambda v)}^{2}}]$ is angular spectrum diffraction formula. It is noted from Eq. (3) that oblique diffraction with any distance is accomplished by incorporating obliquity factor matrix exp [i*k* (*x*cos *α* + *y*cos*β*)] into numerical diffraction calculation. In particular, the incident angle *α*, *β*, *γ* should meet the following condition

4 $${\cos }^{2}\,\alpha +{\cos }^{2}\,\beta +{\cos }^{2}\,\gamma =1.$$

**Figure 2:**
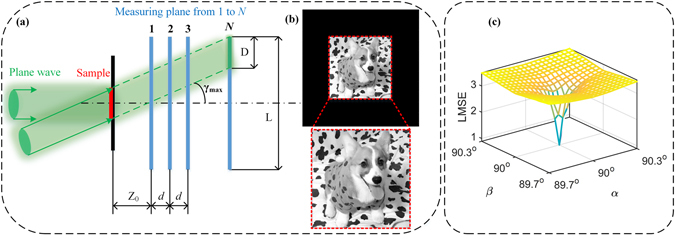
The image reconstruction test using APR: (**a**) The diffraction boundary of tilt illumination. (**b**) The ground truth for test. (**c**) The LMSE distribution between ground truth and reconstructed amplitude by APR algorithm under tilt illumination.

Numerical computation of tilt diffraction should pay attention to the diffraction boundary in Fig. 2(a), out of which the received signal in CCD plane is not intact. If the length of receiving plane is L and the diameter of incident light is D, the maximum of incident angle *γ*_max_ should obey

5 $${\gamma }_{{\rm{\max }}}=\arctan [\frac{{\rm{L}}-{\rm{D}}}{2({{\rm{Z}}}_{0}+(N-1)d)}],$$

for preventing the main spectrum from dismissing. After the aforementioned tilt diffraction model is built, axial multi-image phase retrieval with tilt illumination is finished as long as inserting the presupposed obliquity factor matrix and its complex conjugate into the corresponding forward and backward propagation. Here we call APR algorithm with tilt illumination as APRT algorithm for short, which will be used for the image reconstruction task in simulation and experiment.

To quantitatively assess the reconstructed accuracy for phase retrieval, we utilize the logarithm of mean square error (LMSE) between ground truth *A*_*G*_ and retrieved amplitude *A*_*est*_ as a metric function

6 $${\rm{LMSE}}={\rm{lmse}}({A}_{est},{A}_{G})={\mathrm{log}}_{10}\,(\frac{1}{{\rm{M}}\times {\rm{M}}}{\sum _{\forall {m}_{0},{n}_{0}}\Vert {A}_{est}|-|{A}_{G}\Vert }^{2}),$$

where the size of CCD plane is set as M **×** M pixels and the indexes *m*_0_, *n*_0_ ∈ [1, M]. To visualize the influence of tilt illumination on reconstruction for axial phase retrieval, the LMSE distribution with different incident angle (*α*, *β*) is calculated by APR algorithm. The result is pictured in Fig. 2(c). Here the image exhibited in Fig. 2(b) is selected as ground truth. The parameters follow: Z_0_ = 20 mm, *d* = 2 mm, λ = 532 nm, M = 568, *N* = 8, iterative number is fixed at 1000. As is shown in Fig. 2(c), the LMSE distribution near the position (*α* = *β* = 90°) appears to be a sharp pulse, which indicates that axial multi-image phase retrieval is sensitive to tilt illumination. It further implies that obtaining high-quality recovery of object image is difficult from the tilted experimental data.

### Cross-correlation calibration

To mitigate the impact of tilt illumination on axial phase retrieval, we need a reverse deriving method to characterize the incident angle and then retrieve the sample with tilt illumination. The most significant process is to obtain a relative shift of diffraction pattern. Here we choose the peak position corresponding to the maximum of cross-correlation function between the first and each diffraction image as the center (X_*n*_, Y_*n*_) of every diffraction image. The correlation operation is described as

7 $${C}_{1,n}={L}_{1}\ast {L}_{n},$$

where *L*_*n*_ is the diffraction disc of incident light and the symbol ‘*’ denotes cross-correlation operation. Noticeably, the reason why we use the diffraction disc of incident light to derive the peak position is that the intensity of diffraction disc changes uniformly from inside out by contrast with sample’s diffraction image. Thus this character of diffraction disc makes the shifted center traced much easier.

**Figure 3:**
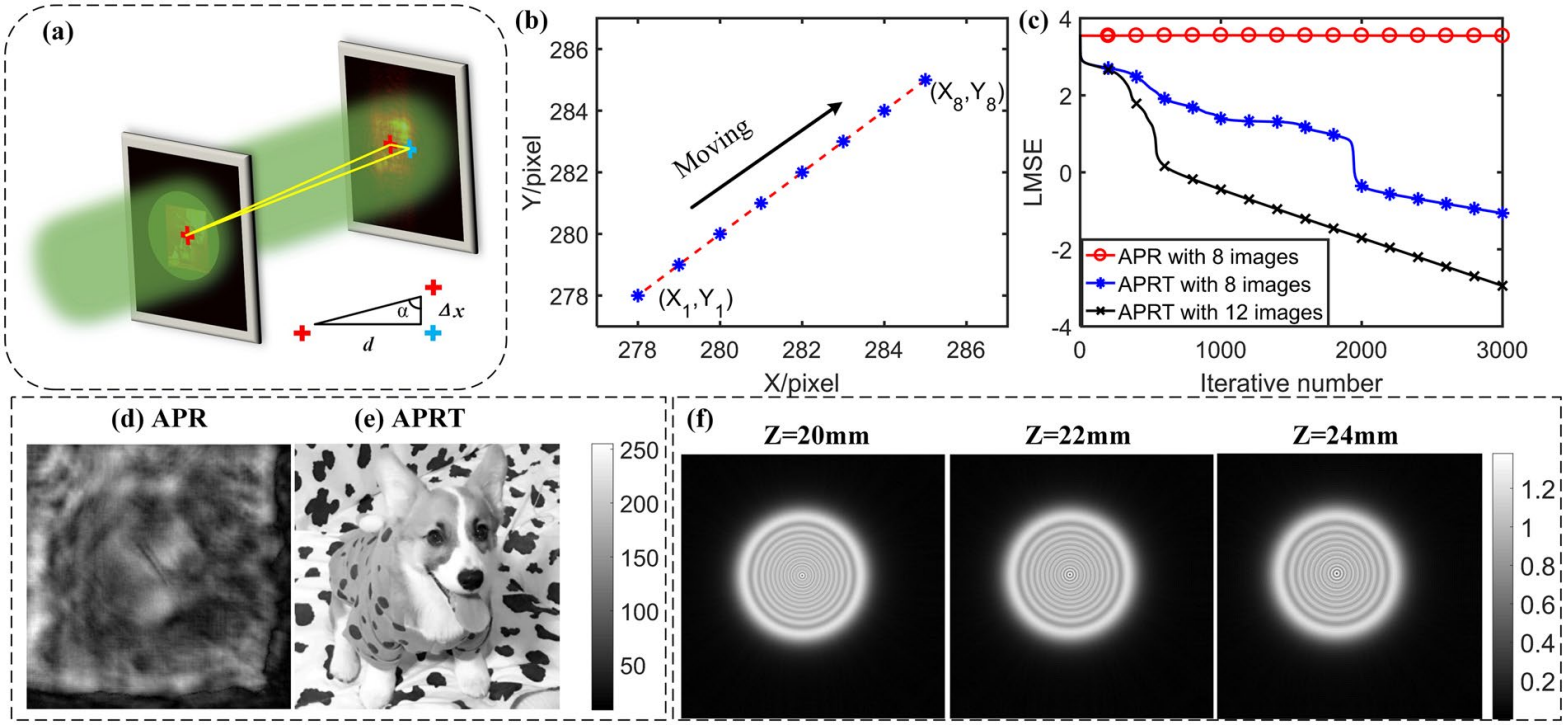
The calibration of tilt illumination for APR algorithm: (**a**) The schematic of cross-correlation calibration. (**b**) The movement of the peak position of cross-correlation. (**c**) The LMSE curve in assigned circumstances. (**d**) and (**e**) denote the uncalibrated and calibrated reconstructed amplitude. (**f**) The diffraction disc of incident light in Z_*n*_ = 20 mm, 22 mm and 24 mm.

The geometrical relation of tilt illumination is given in Fig. 3(a). The angles *α*, *β*, *γ* can be calculated by using Eq. 4 with the displacement Δ*x* and Δ*y*. Utilizing the parameters as same as done in Fig. 2(c) and setting *α* = *β* = 89.8°, a group of diffraction discs are shown in Fig. 3(f). After feeding these images into Eq. (7), the peak position distribution of 8 diffraction discs (step size = 1mm) is pictured in Fig. 3(b). Then, the relative shift derives from

8 $$\{\begin{array}{c}{\rm{\Delta }}{x}_{n}=\varepsilon ({{\rm{X}}}_{n+1}-{{\rm{X}}}_{n}),\\ {\rm{\Delta }}{y}_{n}=\varepsilon ({{\rm{Y}}}_{n+1}-{{\rm{Y}}}_{n}),\end{array}$$

where the bracketed expression is the relative shifted pixels and *ε* is the pixel size of CCD. To get a robust estimation of pattern displacement, we make use of the following equation to acquire the averaged shift as

9 $$\{\begin{array}{c}{\rm{\Delta }}x=\frac{\varepsilon }{N-1}\sum _{n=1}^{N-1}({{\rm{X}}}_{n+1}-{{\rm{X}}}_{n}),\\ {\rm{\Delta }}y=\frac{\varepsilon }{N-1}\sum _{n=1}^{N-1}({{\rm{Y}}}_{n+1}-{{\rm{Y}}}_{n}).\end{array}$$

As is shown in Fig. 3(a), the estimated oblique angle can be solved by

10 $$\{\begin{array}{c}{\alpha }_{est}={\rm{arccot}}({\rm{\Delta }}x/d),\\ {\beta }_{est}={\rm{arccot}}({\rm{\Delta }}y/d).\end{array}$$

Combining the data (Δ*x*, Δ*y*) from Fig. 3(b) with Eq. (10), we can get that *α*_*est*_ = *β*_*est*_ = 89.7983° while *α* = *β* = 89.8°. Although a little bias appears in the guessed angle, the subsequent simulation is to verify the effectiveness of our calibration. Reconstructed images obtained by APR algorithm and APRT algorithm are presented in Fig. 3(d) and (e), respectively. APRT algorithm is achieved on the basis of APR algorithm by adding up the obliquity factor matrix and its complex conjugate into forward and backward propagation at each iteration. It is noted from Fig. 3(d) and (e) that the retrieved image calibrated by *α*_*est*_ and *β*_*est*_ tends to be convergent and clear again. The detailed convergence curves are also depicted in Fig. 3(c), which proves that our cross-correlation calibration is sufficiently feasible even if a little bias exists in the estimated angle.

## Experimental Results

**Figure 4:**
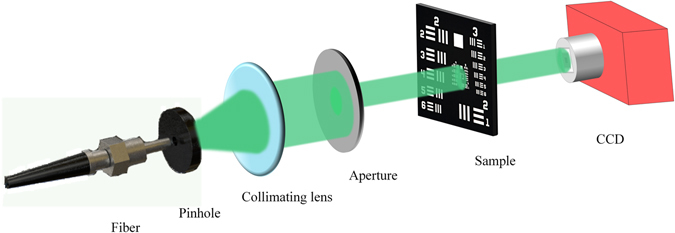
Experiment setup. A green fiber laser at the wavelength of 532 nm passes through pinhole and collimating lens to generate a plane wave. The plane wave shaped by aperture is used to illuminate the resolution chart to form a series of diffraction patterns with the initial distance Z_0_ and interval *d*.

To further exhibit the performance of APRT algorithm with cross-correlation calibration, we apply the above methods into experiment and our experiment setup is displayed in Fig. 4. Here CCD camera (3.1 μm, Point Grey) is mounted on the precision linear stage (M-403, Physik Instrumente Inc.) to generate different diffraction patterns. A fiber laser (532 nm) is shaped by pinhole and collimating lens to form a plane wave. Then, the plane wave passes through an aperture to illuminate the USAF resolution chart for imaging.

**Figure 5:**
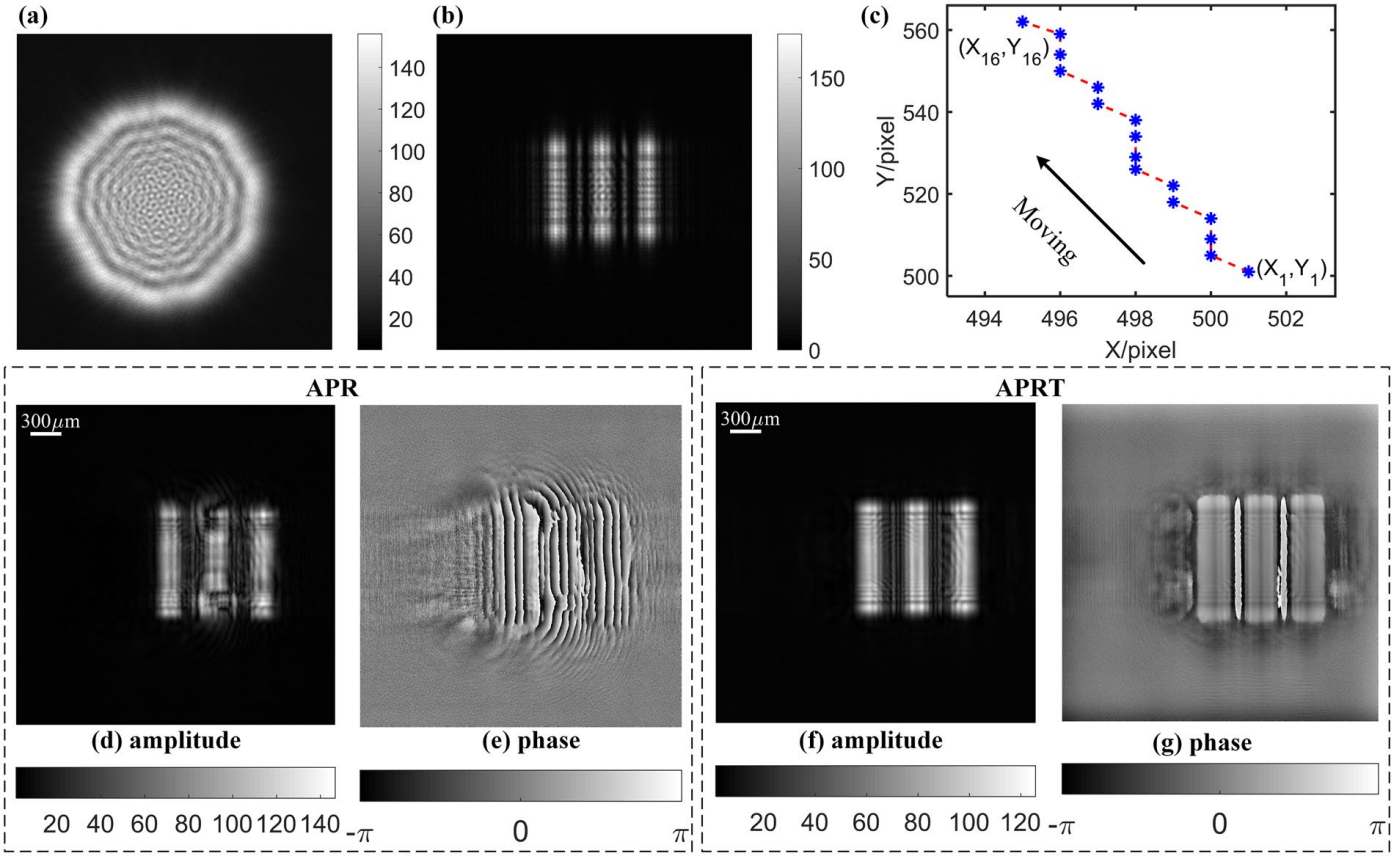
Recorded data and reconstructed complex amplitude in Z_0_ = 50 mm, *d* = 2 mm, *N* = 16, M = 1000: (**a**) Diffraction disc of incident light in Z_1_ = 50 mm. (**b**) Diffraction pattern in Z_1_ = 50 mm. (**c**) The movement of the peak position of cross-correlation; (**d**) and (**e**) are reconstructed amplitude and phase by APR algorithm, respectively; (**f**) and (**g**) are reconstructed amplitude and phase by APRT algorithm, respectively. Here iterative number is equal to 1000.

By adjusting the size of illumination light and setting parameters as follows: Z_0_ = 50 mm, *d* = 2 mm, M = 1000 and iterative number is 1000, a vertical fringe in the resolution chart is illuminated and 16 diffraction patterns such as Fig. 5(b) are measured. The centers of corresponding recorded diffraction discs like as Fig. 5(a) are collected and then Fig. 5(c) is calculated accordingly. Actually, the position distribution in Fig. 5(c) is slightly different from simulation, since background noise affects energy distribution for each diffraction disc. Due to this reason, the operation of average in Eq. 9 is helpful for smoothing this disturbance. The incident angle (*α*_*est*_, *β*_*est*_) is calibrated by virtue of Eqs (7–10) as (89.6388°, 90.0355°).

With the plug-in of estimated incident angle, the target’s amplitude and phase are retrieved by APRT algorithm in Fig. 5(f) and (g), respectively. The shape of vertical fringe in Fig. 5(f) and (g) is easy to be distinguished by contrast with the results from APR method in Fig. 5(d) and (e), which indicates that APRT algorithm with cross-correlation calibration is successful by the modification according to tilt illumination.

**Figure 6:**
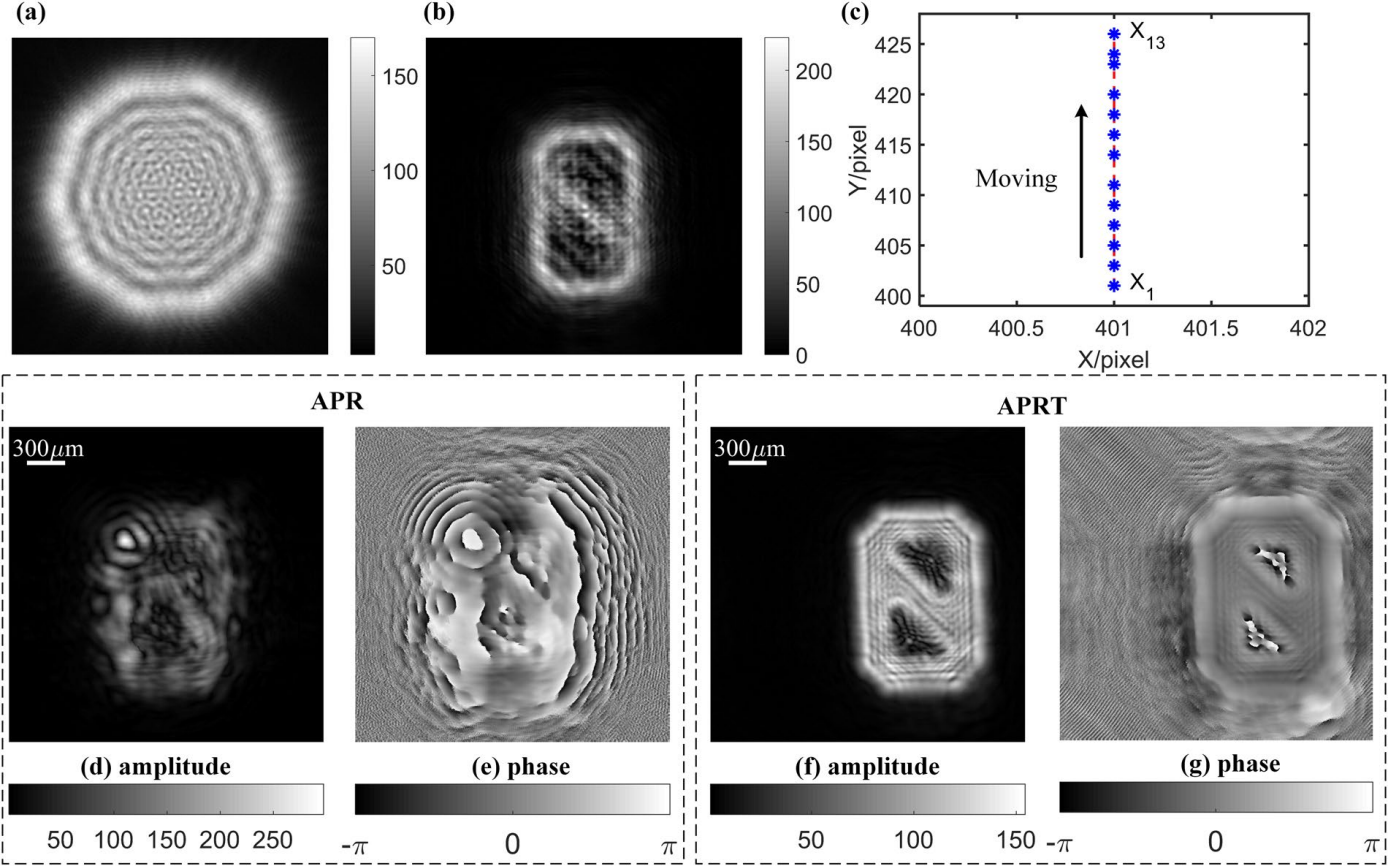
Recorded data and reconstructed complex amplitude in Z_0_ = 60 mm, *d* = 1 mm, *N* = 13, M = 800: (**a**) Diffraction disc of incident light in Z_1_ = 60 mm. (**b**) Diffraction pattern in Z_1_ = 60 mm. (**c**) The movement of the peak position of cross-correlation; (**d**) and (**e**) are reconstructed amplitude and phase by APR algorithm, respectively; (**f**) and (**g**) are reconstructed amplitude and phase by APRT algorithm, respectively. Here iterative number is fixed at 2000.

To validate the performance of our proposed method on complex structure, a number ‘0’ is also used as the target to be retrieved. After selecting different parameters like as: Z_0_ = 60 mm, *d* = 1 mm, M = 800 and iterative number is fixed at 2000, incident angle (*α*_*est*_, *β*_*est*_) is estimated as (89.63°, 90°) and the results are presented in Fig. 6. As for APR algorithm, it suffers from a crushing defeat in the reconstruction. On the contrary, APRT algorithm behaves excellently in terms of the recovery fidelity of both amplitude and phase. Therefore, APRT algorithm along with cross-correlation calibration is useful to eliminate the impact of tilt illumination on axial multi-image phase retrieval. Also, after these calibrations, multi-image phase retrieval based on displacement diversity is also verified here.

## Discussion

**Figure 7:**
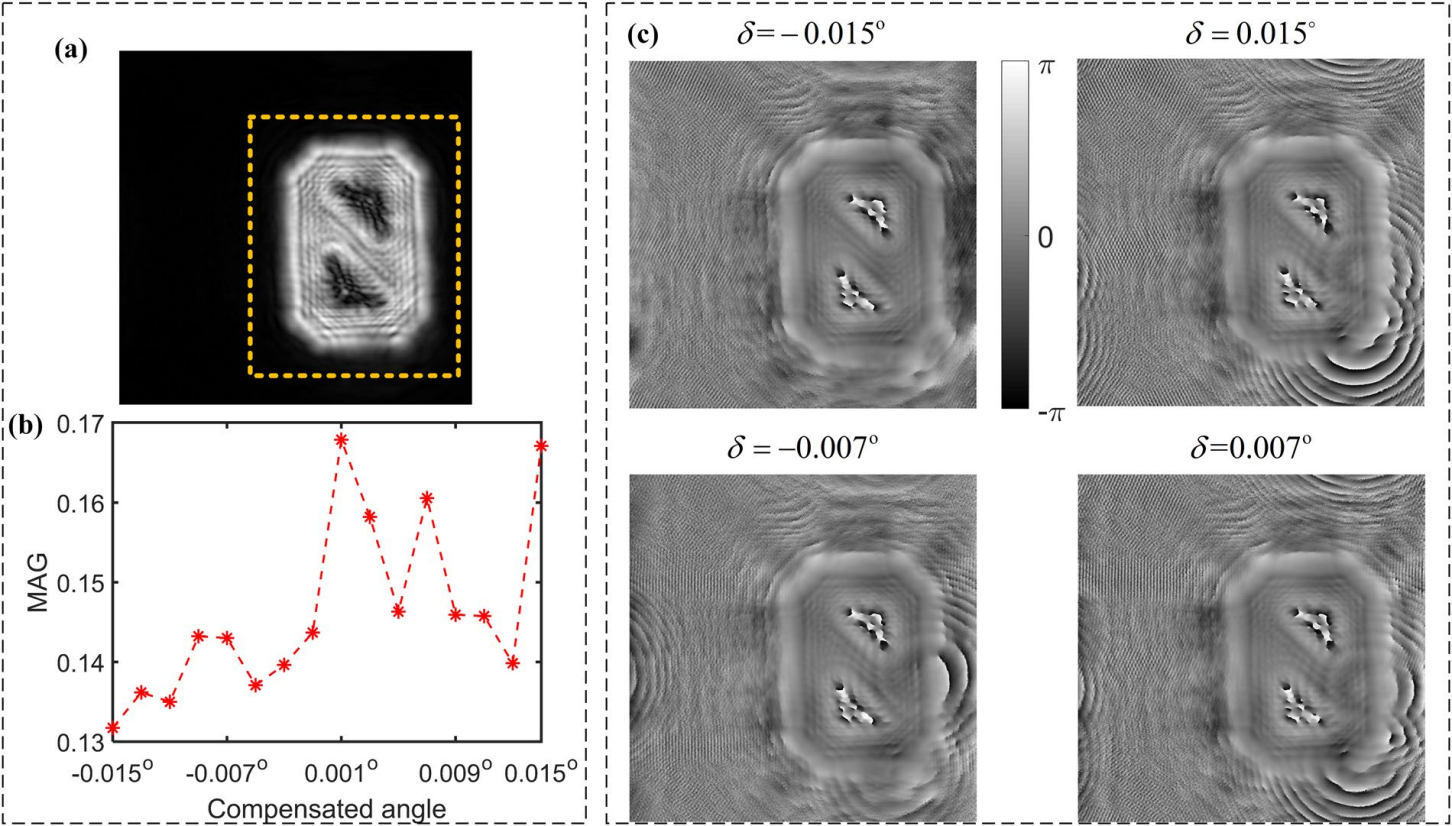
The reconstructed phase with different angle compensation: (**a**) Retrieved amplitude distribution. (**b**) MAG curve, (**c**) Retrieved phase patterns.

In simulation, there is an inevitable angle bias in calibration due to the quantization error. However, the APRT algorithm is still robust enough to retrieve a full complex amplitude at the expense of a large iterative number, which is also demonstrated in experiment. So we assume that if we bring in a compensated angle *δ* on the basic of the estimated angle *α*_*est*_, APRT algorithm may converge quickly. For example, searching for the maximum pairs of cross-correlation, X_1_ = 2, X_2_ = 2, X_3_ = 3, which are hypothetically peak positions, may happen in the case ${{\rm{X}}}_{1}^{^{\prime} }=1.51$, ${{\rm{X}}}_{2}^{^{\prime} }=2.49$, ${{\rm{X}}}_{3}^{^{\prime} }=3.47$. Under this hypothesis, the quantization error happening in Eq. (8) results in the bias of ± 1 pixel. Hence the range of estimated incident angle is immobilized between 89.615° ≤ *α*_*est*_ ≤ 89.645° by inserting this ± 1 pixel into the physical parameters in Fig. 6. To mitigate the quantization error from digital subdivision, we run APRT algorithm with angle compensation *δ* ∈ [−0.015°, 0.015°] and the results are shown in Fig. 7.

Generally, the gradient of phase denotes the magnitude of phase change. Resolution chart is regarded as a sample with constant phase. If reconstructed phase is not convergent, its magnitude will be more fluctuating than convergent case. Thus we can utilize the gradient of phase to indicate the blurs of reconstructed image. Considering that the gradient has both positive and negative magnitude, we use the mean of absolute value of gradient (MAG) as a metric function. The bigger the MAG is, the stronger the fluctuation of reconstructed phase is. Here we choose the section inside yellow dashed rectangle shown in Fig. 7(a) to assess the image quality. After 280 iterations, the MAG curve with the compensated angle of [−0.015°, 0.015°] is illustrated in Fig. 7(b). Four of them are displayed in Fig. 7(c). It is noted that *δ* = −0.015° reaches to the minimum and its reconstructed phase is visually flat and clear. On the contrary, other phase surfaces in Fig. 7(c) still remain to be eddy on the edge. Thus we can find that adding a compensated angle of −0.015° into estimated angle *α*_est_ speeds up the convergence of APRT algorithm with a factor of seven. Hence the APRT algorithm could be applied as the indicator of off-axis in an optical system. After gaining a rough estimated angle from cross-correlation calibration, APRT algorithm with angle compensation is able to measure the oblique angle of illumination light. For coherent diffractive imaging, the resolution is determined by the aperture of CCD camera (2.48 mm, 800 × 800 pixels). With the limitation of 0.061*λ*/NA and sampling theorem, the resolution should be theoretically fixed at ~6 μm in Figs 5 and 6. The shift bias from cross-correlation calibration is ±1 pixel, namely ±3.1 μm. The bias is close to smallest resolution of CDI. To avoid this obstruction, the size of sample is 10 times bigger than the smallest resolution in experiment.

## Conclusions

In this paper, we build the tilt diffraction modality for axial multi-image phase retrieval and prove that axial multi-image phase retrieval is sensitive to tilt illumination. To get a perfect phase retrieval with tilt illumination, we propose a cross-correlation calibration to estimate the oblique angle of illumination light and then use APRT algorithm to reconstruct the full complex amplitude of sample, which is demonstrated in simulation and experiment. Hence the limitation of sensitivity to tilt illumination is overcome in axial multi-image phase retrieval. Also, APRT algorithm can provide a new perspective for numerical off-axis detection. In experimental setups, the absolute on-axis of optical system is almost impossible and difficult to achieve. As a feedback derived method, APRT algorithm with cross-correlation calibration could characterize the oblique status of incident light, which could be a solid and reliable indicator for adjusting an optical system. Generally, this method will benefit other image reconstruction techniques, such as PIE with some off-axis recorded images.

In spite of good performance, the limitations of APRT algorithm with cross-correlation calibration lies in two aspects. First, it is time consuming to compensate the corresponding oblique angle. High-accuracy of angle estimation asks for huge subdivision of compensated range. Second, the MAG is a rough assessment for image quality. For the sample with non-constant phase, determining the blurs of image from different compensated angle needs more robust no-reference image assessment method.
